# Candidate genes for productivity identified by genome-wide association study with indicators of class in the Russian meat merino sheep breed

**DOI:** 10.18699/VJ20.681

**Published:** 2020-12

**Authors:** A.Y. Krivoruchko, O.A. Yatsyk, E.Y. Safaryan

**Affiliations:** All-Russian Research Institute of Sheep and Goat Breeding – Branch of the North Caucasus Federal Agricultural Research Center, Stavropol, Russia; All-Russian Research Institute of Sheep and Goat Breeding – Branch of the North Caucasus Federal Agricultural Research Center, Stavropol, Russia; All-Russian Research Institute of Sheep and Goat Breeding – Branch of the North Caucasus Federal Agricultural Research Center, Stavropol, Russia

**Keywords:** sheep, SNP, genome-wide association study, GWAS, candidate gene, Russian meat merino, овца, однонуклеотидный полиморфизм, полногеномный поиск ассоциаций, полногеномный анализ ассоциаций, ген-кандидат, российский мясной меринос

## Abstract

Genome-wide association studies allow identification of loci and polymorphisms associated with the formation of relevant phenotypes. When conducting a full genome analysis of sheep, particularly promising is the study of individuals with outstanding productivity indicators – exhibition animals, representatives of the super-elite class. The aim of this study was to identify new candidate genes for economically valuable traits based on the search for single nucleotide polymorphisms (SNPs) associated with belonging to different evaluation classes in rams of the Russian meat merino breed. Animal genotyping was performed using Ovine Infinium HD BeadChip 600K DNA, association search was performed using PLINK v. 1.07 software. Highly reliable associations were found between animals belonging to different evaluation classes and the frequency of occurrence of individual SNPs on chromosomes 2, 6, 10, 13, and 20. Most of the substitutions with high association reliability are concentrated on chromosome 10 in the region 10: 30859297–31873769. To search for candidate genes, 15 polymorphisms with the highest association reliability were selected (–log10(р) > 9). Determining the location of the analyzed SNPs relative to the latest annotation Oar_rambouillet_v1.0 allowed to identify 11 candidate genes presumably associated with the formation of a complex of phenotypic traits of animals in the exhibition group: RXFP2, ALOX5AP, MEDAG, OPN5, PRDM5, PTPRT, TRNAS-GGA, EEF1A1, FRY, ZBTB21-like, and B3GLCT-like. The listed genes encode proteins involved in the control of the cell cycle and DNA replication, regulation of cell proliferation and apoptosis, lipid and carbohydrate metabolism, the development of the inflammatory process and the work of circadian rhythms. Thus, the candidate genes under consideration can influence the formation of exterior features and productive qualities of sheep. However, further research is needed to confirm the influence of genes and determine the exact mechanisms for implementing this influence on the phenotype.

## Abstract (ru)

Полногеномный поиск ассоциаций позволяет идентифицировать локусы и отдельные полиморфизмы, связанные с формированием интересующих фенотипов. При полногеномном анализе у овец особо перспективным представляется изучение особей, отличающихся выдающимися показателями продуктивности – выставочных животных, представителей класса «суперэлита». Целью настоящего исследования явилось выявление новых генов-кандидатов хозяйственно ценных признаков на основе поиска однонуклеотидных полиморфизмов, ассоциированных с принадлежностью к различным бонитировочным классам, у баранов породы российский мясной меринос. Генотипирование животных выполнено с использованием ДНК-биочипов Ovine Infinium HD BeadChip (600K), поиск ассоциаций – с использованием программного обеспечения PLINK v. 1.07. Выявлены высокодостоверные ассоциации между принадлежностью животных к различным бонитировочным классам и частотой встречаемости отдельных однонуклеотидных полиморфизмов на хромосомах 2, 6, 10, 13 и 20. Большая часть замен с высокой достоверностью ассоциаций сконцентрирована на хромосоме 10 в области 30859297–31873769. Для поиска генов-кандидатов отобрано 15 полиморфизмов с наибольшей достоверностью ассоциаций (–log10(р) > 9). Определение местоположения анализируемых однонуклеотидных полиморфизмов относительно новейшей аннотации Oar_rambouillet_v1.0 позволило выявить 11 генов-кандидатов, предположительно, связанных с формированием комплекса фенотипических признаков животных выставочной группы: RXFP2, ALOX5AP, MEDAG, OPN5, PRDM5, PTPRT, TRNAS-GGA, EEF1A1, FRY, ZBTB21-like и B3GLCT-like. Перечисленные гены кодируют белки, вовлеченные в контроль клеточного цикла и репликации ДНК, регуляцию пролиферации и апоптоза клеток; участвующие в липидном и углеводном обменах, развитии воспалительного процесса и работе циркадных ритмов. Благодаря этому рассматриваемые гены-кандидаты могут влиять на формирование экстерьерных особенностей и продуктивные качества овец. Однако необходимы дальнейшие исследования, направленные на подтверждение влияния генов и определение точных механизмов этого воздействия на фенотип.

## Introduction

Genome-wide association study (GWAS) is a modern and powerful tool for identifying loci and individual polymorphisms associated with economically important traits in various species of productive animals (Georges et al., 2019). Loci associated with reproductive qualities (Abdoli et al., 2019), resistance to parasitic diseases (Yan et al., 2017), indicators of wool (Wang Z. et al., 2014), milk (Garcia-Gámez et al., 2012) and meat productivity (Rovadoscki et al., 2018; Zhang T. et al., 2019) were identified in the sheep genome using GWAS tools.

Most of these studies identify associations with a specific performance trait characteristic of the breed under study. In our opinion, the search for loci associated not with individual parameters of productivity, but with a complex of phenotypic characteristics that determine the breeding value and class of sheep during grading is of particular interest. The division of sheep into classes is carried out according to the aggregate level of wool and meat productivity, constitutional characteristics and the degree of compliance with the breed standard. The most valuable is the study of rare genotypes of outstanding representatives of the breed – exhibition animals, according to the results of the appraisal assigned to the superelite class. Identification of genetic markers of class opens up opportunities for genetic assessment, selection of highly productive animals and optimal selection of parental pairs capable of transferring their economically valuable characteristics to offspring

The most common approach of GWAS is to search for associations with the analyzed quantitative trait (for example, live weight) (Gudmundsdottir, 2015). But in the case of a search for associations with belonging to the super-elite class associated with a relatively small sample size, it is advisable to use a non-quantitative analysis approach of the case-control type. In such an analysis, an individual carrying the phenotypic trait of interest gets into the case group, and the individual without the qualities of interest into the control group (Gudmundsdottir, 2015). Previously, non-quantitative analyzes have been successfully performed in sheep for white wool/ non-white wool traits (Kijas et al., 2013), multiple pregnancy/ non-multiple pregnancy (Xu et al., 2018), high muscle mass/ low muscle mass (Gudmundsdottir, 2015). If associations with class are identified during GWAS, the phenotype of an animal of the super-elite class can be designated as “case”, and the phenotype of the main herd as “control.”

It seems promising to conduct a search for genome-wide associations in animals of the Russian meat merino breed, which combines high wool and meat productivity. Sheep of the Russian meat merino breed exceed the current minimum requirements for sheep of the meat-wool production type in terms of live weight and shearing of washed wool. The average live weight of stud rams is 107 kg, and the live weight of super-elite rams reaches 121 kg (Amerkhanov et al., 2018). Animals are characterized by a strong constitution, hornless rams and ewes, thick, thin and even hair, high vigor and pronounced meat forms (Selionova et al., 2017).

In this regard, the purpose of this study was to identify new candidate genes for economically valuable traits based on the search for single nucleotide polymorphisms (SNPs) associated with belonging to different grading classes in Russian meat merino breed. 

## Materials and methods

The studies were carried out on the basis of the laboratories of the All-Russian Research Institute of Sheep and Goat Breeding – branch of the North Caucasus Federal Scientific Agricultural Center (Stavropol, Russia), the Skolkovo Institute of Science and Technology “Skoltech” (Moscow, Russia), the Scientific Diagnostic and Veterinary Medicine Center of the Stavropol State Agrarian University (Stavropol, Russia), stud farm “Vtoraya Pyatiletka” of the Stavropol region (Russia).

**Fig. 1.**
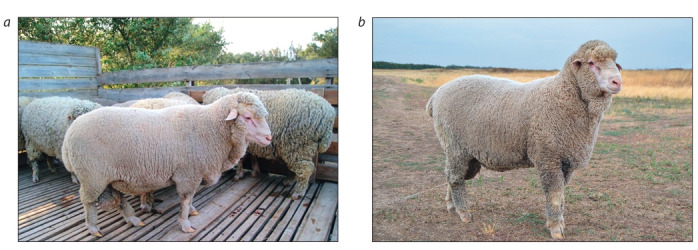
Russian meat merino sheep breed: а – phenotype “case”, b – phenotype “control.

The object of the study was the Russian meat merino sheep, 12 months old (n = 54), belonging to the breeding group. Based on the results of the assessment carried out, 49 rams were assigned the elite class, they made up the control group (Fig. 1, a). Five animals were characterized as super elite. The latter, as outstanding individuals, were selected into the group of exhibition animals and were characterized as animals with the “case” phenotype parameter (Fig. 1, b). All rams were clinically healthy.

Quality control of genotyping

Quality control of genotyping was carried out using the PLINK v. 1.07 software (Purcell et al., 2007). The data processing included samples with an indicator of the number of detected SNPs (call rate) greater than 0.95. SNPs with no chromosomal or physical localization, with the minor allele frequency less than 0.01, and the missing genotypes frequency (missing genotype) more than 0.1 were excluded from the analysis. The value p = 0.0001 was used as the threshold value according to the Hardy–Weinberg equilibrium criterion by the Fisher method. With a positive result, 54 samples passed the quality control of genotyping (5 samples of the “case” phenotype, 49 samples of the “control” phenotype). From 606,006 SNPs, 521,829 polymorphisms were used for further analysis.

Genetic and statistical analysis

A genome-wide search for associations was performed using the PLINK v. 1.07 software, the assoc function (Purcell et al., 2007) based on the assessment of the significance of the SNP influence on the attribution class. To confirm the significance of differences in multiple comparisons, the p-score with Bonferroni’s correction was used. Visualization and plotting were performed using the QQman package in the R programming language. The search for candidate genes was carried out among the nearest genes located at a distance not exceeding 200,000 bp from SNP, which showed significant differences in the occurrence among animals of the studied groups. In connection with the appearance of updated assemblies of the sheep genome containing updated information on the location and sequences of encoded genes, the location of analyzed SNPs was estimated using the current annotation Oar_rambouillet_v1.0. Gene annotation was performed using the tools of the National Center for Biotechnology Information (https:// www.ncbi.nlm.nih.gov).

## Results

**Fig. 2.**
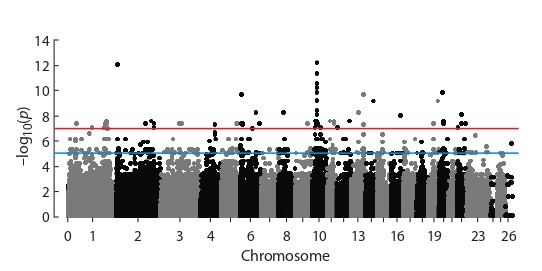
Manhattan plot of the results of the GWAS with –log10(p) values for investigated SNP. Here and also in Fig. 4 the lower line indicates the threshold of the expected significance of differences at the value –log10(p) = 5, the upper line indicates the threshold of high significance of differences at the value of –log10(p) = 7.

As a result of a genome-wide associations search between the frequency of occurrence of individual SNPs and the animals belonging to the exhibition group, more than 50 single nucleotide substitutions were identified that passed the confidence threshold, determined taking into account the Bonferroni correction. The threshold for –log10( p) values was 0,95*7, the top line in the Manhattan plot (Fig. 2).

**Fig. 3.**
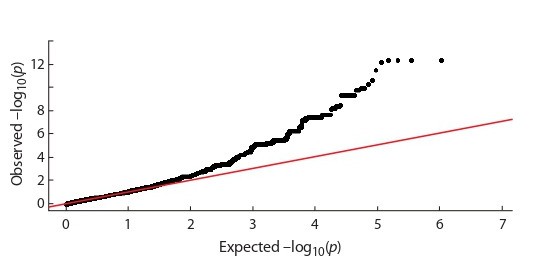
Quantile-quantile plot for the probabilities of the distribution of the validity of SNP estimates throughout the genome.

The results of the differences significance distribution assessment for 26 chromosomes are shown in the quantilequantile plot. Beginning with –log10( p) > 2, a deviation from the theoretically expected distribution is observed if the null hypothesis is confirmed (Fig. 3). 

**Fig. 4.**
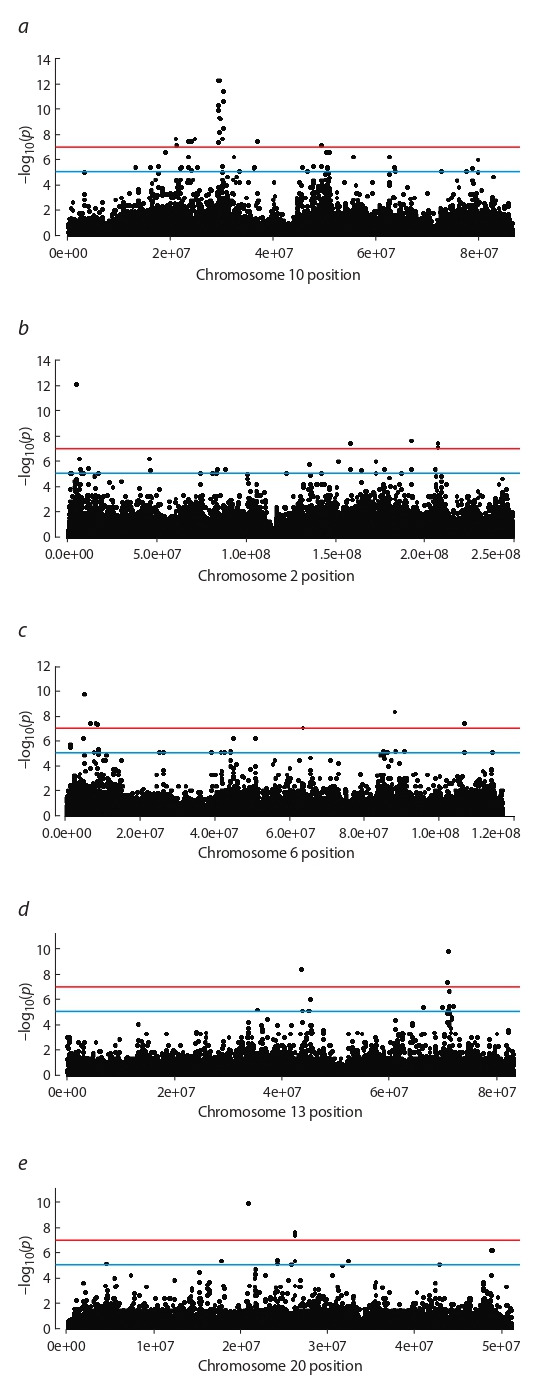
Manhattan plot that shows the results of the GWAS with –log10(p) values for investigated SNPs on chromosomes 10, 2, 6, 13 and 20.

The largest number of significant associations was found for polymorphisms located on chromosome 10 (Table). The Manhattan plot shows that the substitutions with the highest confidence value are located relatively close to each other (Fig. 4, a). 

**Table 1.**
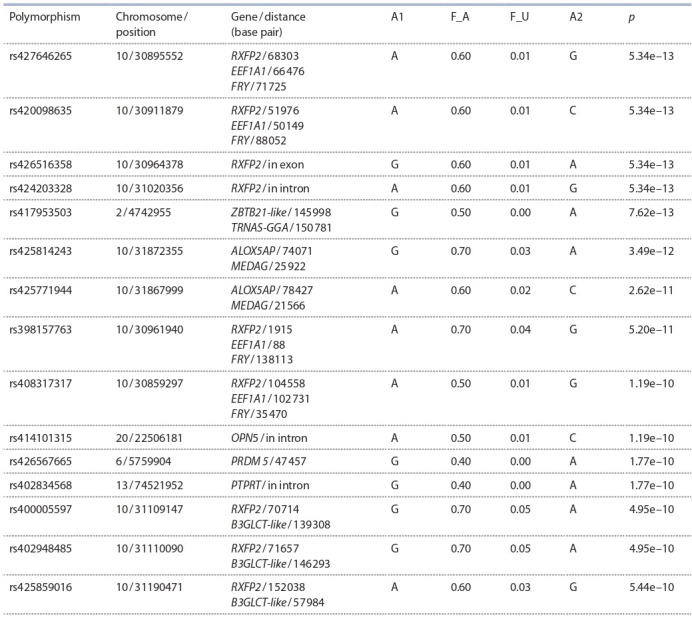
Characterization of the SNP with the highest reliability indicators of association with the exhibition group of animals during the GWAS Notе. A1 – minor allele; A2 – major allele; F_A – frequency of minor allele in the exhibition group of animals; F_U – frequency of minor allele in the selection group.

In a more detailed analysis of their localization, it wasfound that most of them are concentrated in the region with coordinates from 30859297 to 31873769 1 Mb in length, which includes the sequences of 9 different genes. Also, a high reliability of associations was revealed for SNPs located on chromosomes 2, 6, 13, 20. However, on these chromosomes it was not possible to identify areas with a high concentration of reliable associations, since the substitutions are located at a significant distance from each other (Fig. 4, b–e).

To search for candidate genes, 15 polymorphisms were selected with the highest reliability of associations (–log10( p) > 9), among them one missense mutation in the exon, two substitutions located in gene introns, and eleven substitutions located in intergenic areas (see Table).

High reliability of associations was found for the substitutions rs426516358 and rs424203328 located in exon 18 and intron 1–2 of the RXFP2 gene, as well as for substitutions located in adjacent intergenic regions. So, the substitutions rs427646265, rs420098635, rs398157763, and rs408317317 are localized in the region between the RXFP2 and FRY genes. Substitutions rs400005597, rs402948485 and rs425859016 – between genes RXFP2 and B3GLCT-like. The rs425814243 and rs425771944 polymorphisms are located in the region between the ALOX5AP and MEDAG genes. The single nucleotide substitution rs417953503 is located in the intergenic region, practically at an equal distance from the ZBTB21- like pseudogen and the gene encoding tRNA TRNAS GGA. The rs426567665 polymorphism is located in the intergenic region, at a distance of 47 kbp from the PRDM5 gene. The rs402834568 substitution is located in intron 5–6 of the PTPRT gene. The rs414101315 polymorphism, highly reliably associated with the super-elite group of animals, is located in intron 4–5 of the OPN5 gene.

## Discussion

In the presented work, to identify SNPs associated with performance indicators, a non-quantitative analysis of the casecontrol type was used, based on comparing the frequency of SNP occurrence in rams of different grading classes, differing in breeding value, wool and meat productivity. A similar approach was previously used to analyze the frequency of SNP occurrence in rams with high and low muscle mass, differing in the level of meat productivity. At the same time, 13 candidate genes for muscle growth and meat productivity were identified on ten different chromosomes (Gudmundsdottir, 2015). As a result of our work, 11 candidate genes were identified on 5 chromosomes, presumably associated with the formation of a complex of phenotypic traits demonstrated by animals of the super-elite class.

Chromosome 10. According to the results of GWAS, one of the most promising candidate genes, probably associated with the belonging of animals to different grading classes in Russian meat merino sheep, is the gene RXFP2 (relaxin family peptide receptor 2), the gene for the relaxin family peptide receptor. The RXFP2 receptor mediates the action of relaxin and insulin-like peptides, which play an important physiological role in the functioning of the reproductive and cardiovascular systems (Scott et al., 2012). The expression level of RXFP2 positively correlates with the concentration of testosterone in the blood (Johnston et al., 2011). In sheep, RXFP2 is a marker gene for predicting the type and length of horns (Dominik et al., 2012; Wiedemar, Drögemüller, 2015; Duijvesteijn et al., 2018). Thus, some substitutions associated, according to the results of our studies, with the phenotype of the exhibition animal, were previously proposed to predict the phenotype of the horn. Substitution of rs426516358 in exon 18 of the RXFP2 gene leads to a change in the encoded amino acid (p.Leu687Phe). According to the results of studies by N. Duijvesteijn et al. (2018), male merino sheep with the GG genotype for the replacement rs426516358 will always be hornless. The substitution rs408317317 has been proposed as a marker of the hornless phenotype for Australian merino sheep (Dominik et al., 2012); its relationship with the type, length, and circumference of the horn base in wild sheep Soay has been revealed (Johnston et al., 2013). The rs398157763 substitution is also associated with horn characteristics in wild Soay sheep (Johnston et al., 2011). There is evidence that, by affecting the formation of horns, polymorphism of the RXFP2 gene and adjacent regions also affects reproductive success and survival in wild sheep. Most interestingly, in our study, the polymorphism of the RXFP2 gene and its flanking regions was associated with the conformation characteristics of hornless rams.

Promising candidate genes are also genes located in relative proximity to the RXFP2 gene and polymorphisms with a high reliability of associations: genes EEF1A1, FRY, and B3GLCT-like. The EEF1A1 gene (elongation factor 1-alpha 1, LOC101110773) is 58 bp away from the RXFP2 gene in its 3′-flanking region. In humans, the EEF1A1 gene is responsible for the enzymatic delivery of aminoacyl tRNAs to the ribosome and is involved in the maintenance of cell homeostasis as a regulator of proliferation and apoptosis (Dapas et al., 2020). The substitution rs398157763 considered in sheep as a marker of polledness is located at a distance of 88 bp from the EEF1A1 gene. The FRY gene ( protein furry homolog, LOC101110521) encodes a protein that interacts with protein kinases in signaling pathways and induces changes in gene expression. The FRY protein activates the Hippo/Yap pathway, which controls the size of internal organs in animals by regulating cell proliferation and apoptosis (Liu et al., 2019). The B3GLCT-like gene (beta-1,3-glucosyltransferase-like, LOC114116650) is a homologue of the B3GLCT gene, which encodes an enzyme involved in protein metabolism and glycosylation (Weh et al., 2017).

The ALOX5AP and MEDAG genes are located in relative proximity to the substitutions with high confidence in the associations rs425814243 and rs425771944. The ALOX5AP gene (arachidonate 5-lipoxygenase activating protein) encodes a protein essential for the synthesis of leukotrienes. It belongs to the family of non-heme iron oxygenases involved in the production and metabolism of fatty acid hydroperoxidases. In sheep, an association of polymorphisms located in the flanking region of the ALOX5AP gene with the fat tail phenotype was revealed (Moioli et al., 2015). For fat tailed sheep, the gene was also considered to be associated with climate adaptation (Mastrangelo et al., 2019). MEDAG (mesenteric estrogen dependent adipogenesis) is an adipogenic gene capable of stimulating the differentiation of preadipocytes into adipocytes, increasing the lipid content and the rate of glucose uptake by cells. It is expressed predominantly in the cells of the visceral fat depot (Zhang H. et al., 2012)

Chromosome 2. The rs417953503 polymorphism identified in the super-elite class is located between the ZBTB21-like pseudogene (LOC101117056, zinc finger and BTB domaincontaining protein 21-like) and the TRNAS GGA transfer RNA gene (transfer RNA serine, anticodon GGA). The product of the true gene ZBTB21 is a negative regulator of transcription for genes that control cell division and DNA replication (Wang J. et al., 2005). In humans, a connection between the ZBTB21 gene polymorphism and the indicator of physical performance was revealed. Interesting that the ZBTB21 gene has been proposed as a candidate gene associated with tenderness in beef (Boudon et al., 2020). Transport RNA genes ensure the delivery of activated amino acid residues to the ribosome and their incorporation into the synthesized protein chain. The sheep genome contains 120 copies of the TRNAS-GGA gene. In merino sheep, a copy of the TRNAS-GGA gene located on chromosome 6 has been proposed as a candidate gene associated with body weight at birth (Dakhlan et al., 2018). In cattle, according to the results of GWAS, polymorphisms located in the flanking regions of the TRNAS-GGA genes on chromosomes 6 and 24 are associated with live weight at birth (Edea et al., 2018) and sperm viability (Kaminski et al., 2016)

Chromosome 6. The closest candidate gene with respect to the rs426567665 substitution found in the animals of the exhibition group is the PRDM5 gene (PR/SET domain 5), which encodes a DNA-binding transcription factor that affects the functioning of hematopoietic and microRNA genes. The PRDM5 gene regulates the intensity of synthesis of proteins involved in the development and maintenance of fibrillar collagens, connective tissue components, and molecules that regulate cell proliferation, differentiation, migration, and adhesion, including the transforming growth factor beta-2 (Burkitt Wright et al., 2011).

Chromosome 13. The rs402834568 substitution was found in the intron region of the PTPRT ( protein tyrosine phosphatase receptor type T) gene, which encodes a protein from the tyrosine phosphatase family that regulates the mitotic cycle, as well as cell growth and differentiation. The PTPRT gene is expressed in the cells of the nervous system and regulates the development of neurons (Lee, 2015). In farm animals, a connection between the PTPRT gene polymorphism and resistance to some bacterial and parasitic infections was revealed. In goats, polymorphism is associated with resistance to brucellosis (Rossi et al., 2017), in cattle, with resistance to tuberculosis (Bermingham et al., 2014), in Romney sheep, with resistance to invasion by gastrointestinal nematodes (Yan et al., 2017). 

Chromosome 20. The OPN5 gene (opsin 5) is expressed in the retina, skin, brain and spinal cord. It encodes the UVsensitive photopigment neuropsin, which is involved in the regulation of circadian rhythms (Buhr et al., 2019). We propose the OPN5 gene as a candidate gene, since its intron contains the SNP rs414101315 with high reliability of associations. 

## Conclusion

In the course of the work done, highly reliable associations were revealed between the belonging of animals to different grading classes and the frequency of occurrence of individual SNPs on chromosomes 2, 6, 10, 13 and 20. Determination of the location of analyzed SNPs relative to the latest annotation Oar_rambouillet_v1.0. made it possible to identify 11 candidate genes, presumably associated with the formation of a complex of phenotypic traits of animals of the exhibition group: RXFP2, ALOX5AP, MEDAG, OPN5, PRDM5, PTPRT, TRNAS-GGA, EEF1A1, FRY, ZBTB21-like, B3GLCT-like. These genes encode proteins with a number of important biological functions involved in the control of the cell cycle and DNA replication, regulation of cell proliferation and apoptosis, involved in lipid and carbohydrate metabolism, the development of the inflammatory process, and the work of circadian rhythms. Due to this, the candidate genes under consideration can influence the formation of conformational characteristics and productive qualities of sheep. However, further research is needed to confirm the effect of genes and to determine the exact mechanisms of this effect on the phenotype. 

## Conflict of interest

The authors declare no conflict of interest.
